# Supplementary material for: Telomerase modRNA Offers a Novel RNA‐Based Approach to Treat Human Pulmonary Fibrosis
Source: Aging Cell. 2025 Sep 20;24(11):e70240. doi: 10.1111/acel.70240 (PMC12608093; doi:10.1111/acel.70240)
Supplement: Supplementary file 1 — Figure S1: Dose‐dependent increase of hTERT mRNA and telomerase activity in vitro after modRNA hTERT transfection in MRC‐5 cells. (A) Validation of the hTERT and GFP modRNA product size after IVT in 1.5% denatured gel. ModRNA GFP is generated by substituting hTERT ORF with the GFP coding sequence. (B) Images showing the successful transfection of modRNA GFP 2 μg/mL and stable GFP expression for until 48 h and a significant decline at 120 h in MRC‐5 cells. (C) Fold change of hTERT mRNA expression after transfection with modRNA hTERT in a concentration range of 0.25–2 μg/mL compared to modRNA control (modRNA GFP 2 μg/mL) after 24 and 48 h in MRC‐5 cells (n = 3). (D) TRAP assay revealed telomerase activity after 24 h modRNA hTERT transfection in MRC‐5 cells. Positive (pos.) control = HEK293 cells, negative (neg.) control = HUVEC cells, LB = lysis buffer, 1 = lysate, Δ = heat‐inactivated lysate. (E) Quantification of TRAP assays in MRC‐5 (n = 3). *p < 0.05; ***p < 0.001; Two‐way ANOVA, Dunnett's multiple comparisons test. Figure 2: Repetitive treatment of modRNA hTERT increase proliferation capacity and decrease expression of senescence‐related marker in ATII cells. (A) Images showing successful transfection of modRNA GFP 2 μg/mL in ATII cells as observed by GFP expression in 24 an 48 h post transfection. (B) Telomere qFISH analysis demonstrated no telomere elongation after 48 and 96 h modRNA hTERT treatment in ATII cells (n ≥ 84 nuclei per group out of 3 biological replicates were imaged) modRNA GFP is used as the modRNA control. (C) Representative brightfield images on the day of passaging in ATII cells of modRNA control compared to 2× treatment modRNA hTERT. (D) A decreasing trend of senescence‐related markers (CDKN2A, CDKN1A, TP53) was detected after two doses of modRNA hTERT treatment (n = 4). *p < 0.05; One‐way ANOVA or Two‐way ANOVA, Dunnett's or Sidak's multiple comparisons test. Figure 3: Validation of circular RNA. (A) Validation of the size of produced linear [file ACEL-24-e70240-s001.docx]

# **Supplementary File**

## **Telomerase modRNA offers a novel RNA-based approach to treat pulmonary fibrosis**

Jia Li Ye^1,2^, Klaudia Grieger^3,4^, Dongchao Lu^1,5^, Christina Brandenberger^6,7^, Malte Juchem^1,3^, Maria Jordan^3,8^, Lea Oehlsen^1,3^, Patrick Zardo^4,9^, Christopher Werlein^4,10^, Christina Hesse^3,4,8^, Katherina Sewald^3,4,8^, Sandy Tretbar^8,11,12^, Thomas Thum^1,2^, Shambhabi Chatterjee^1,3,8^*, Christian Bär ^1,2,3,8^*

# **Supplementary Methods**

### Quantitative fluorescence *in situ* hybridization (qFISH)

The protocol was adapted from a previously described protocol (O’Sullivan et al., 2005). First cultured cells were washed with PBS and then 150,000-300,000 cells were spotted onto SuperFrost microscope slides (J1800AMNZ Epredia) using a Thermo Shandon Cytospin 3 centrifuge. The slides were stored at 4 °C until all samples were collected for staining. For the staining, the slides were washed twice with PBS for 15 min and then fixed in 4 % PFA in PBS for 2 min. After washing the slides thrice with PBS for 5 min, slides were incubated with pre-warmed pepsin in a 37 °C water bath for 10 min. Followed by washing twice in PBS and again fixed in 4 % PFA for 2 min. Afterwards slides were washed thrice in PBS for 5 min. To dehydrate the cells, slides were incubated in different EtOH concentrations (70 %, 90 % and 100 %) for 5 min each continued by air-drying until no drops were visible. 15-30 µl of Telomerase PNA probe mix (10 mM TrisCl pH 7, 25 mM MgCl_2_, 9 mM citric acid, 82 mM Na_2_HPO_4_, 7 % deionized formamide, 0.25 % blocking reagent and 0.5 mg/mL Telomeric PNA probe) were spotted onto the slides and sealed with cover slips. These slides were incubated at 85 °C for exactly 3 min. Subsequently, the slides were incubated in a dark and wet chamber for around 4 h. Next, the cover slip was removed and washed twice for 15 min each in 10 mM TrisCl pH 7.2, 0.1 % BSA in H_2_O and 70 % formamide under vigorous shaking (550 rpm). Next, slides were washed thrice for 5 min with TBS-T and once for 5 min with PBS. The slides were incubated for 20 min with 1:1,000 Hoechst in the dark and then washed thrice for 5 min with PBS. In the end, the slides were air-dried and around 10 µL Prolong^TM^ Gold Antifade Mountant (P10144 Thermo Fisher Scientific) was dropped onto the slides and sealed with cover slips. The slides were stored in the dark at 4 °C. The images were acquired using 63X oil objective and additional 2X zoom with 405 nm and 561 nm laser in the confocal microscope Zeiss LSM 780. Image analysis was performed using our own software programmed for quantification of the digitized fluorescent signals. The software is based on the principals of the open source Telometer Plugin. The software was developed in C# with the integrated development environment Visual Studio Community Edition 2019 under Microsoft .NET and supplemented with OpenCV routines via the EmguCV.NET wrapper (EmguCV 2023).

### Isolation of genomic DNA and relative telomere length measurement by qPCR

PCLS genomic DNA (gDNA) was isolated using the NucleoSpin Tissue MiniKit (740471.50 Macherey-Nagel). All gDNA samples (10 ng/µL) were used for the real-time PCR, which was performed using the iQ SYBR Green Supermix (1708880 BioRad) on QuantStudio™ 5 Real-Time PCR System (Thermo Fisher Scientific) with following protocol: 95 °C incubation for 10 min and then 35 cycles of 95 °C for 15 s, 54 °C for 120 s and 72 °C for 15 s. Telomere length was measured as previously described (Jahn et al., 2025). Two sets of primers were used, namely Telomere primer pair (fw: CGGTTTGTTTGGGTTTGGGTTTGGGTTTGGGTTTGGGTT; rev: GGCTTGCCTTACCCTTACCCTTACCCTTACCCTTACCCT) and the single copy primer pair amplifying the acidic ribosomal phosphoprotein PO (36B4) gene (fw: CAGCAAGTGGGAAGGTGTAATCC; rev: CCCATTCTATCATCAACGGGT

ACAA). The standard curve of the reference human gDNA (11691112001 Roche) was used as control for ensuring efficient qPCR amplification. The final relative telomere length for each sample was calculated based on the ratio of Telomere (T) and 36B4 (S) amplification.

# **Supplementary Figures**

### Supplementary Figure 1| Dose-dependent increase of hTERT mRNA and telomerase activity *in vitro* after modRNA hTERT transfection in MRC-5 cells.

**A** Validation of the hTERT and GFP modRNA product size after IVT in 1.5 % denatured gel. ModRNA GFP is generated by substituting hTERT ORF with the GFP coding sequence. **B** Images showing the successful transfection of modRNA GFP 2 µg/mL and stable GFP expression for until 48 h and a significant decline at 120 h in MRC-5 cells. **C** Fold change of hTERT mRNA expression after transfection with modRNA hTERT in a concentration range of 0.25 – 2 µg/mL compared to modRNA control (modRNA GFP 2 µg/mL) after 24 h and 48 h in MRC-5 cells (n = 3). **D** TRAP assay revealed telomerase activity after 24 h modRNA hTERT transfection in MRC-5 cells. Positive (pos.) control = HEK293 cells, negative (neg.) control = HUVEC cells, LB = lysis buffer, 1 = lysate, Δ = heat inactivated lysate. **E** Quantification of TRAP assays in MRC-5 (n=3). *p<0.05; ***p<0.001; Two-way ANOVA, Dunnett's multiple comparisons test.

### Supplementary Figure 2| Repetitive treatment of modRNA hTERT increase proliferation capacity and decrease expression of senescence-related marker in ATII cells.

**A** Images showing successful transfection of modRNA GFP 2 µg/mL in ATII cells as observed by GFP expression in 24 h an 48 h post transfection. **B** Telomere qFISH analysis demonstrated no telomere elongation after 48 h and 96 h modRNA hTERT treatment in ATII cells (n ≥ 84 nuclei per group out of 3 biological replicates were imaged) modRNA GFP is used as the modRNA control. **C** Representative brightfield images on the day of passaging in ATII cells of modRNA control compared to 2x treatment modRNA hTERT. **D** A decreasing trend of senescence-related markers (CDKN2A, CDKN1A, TP53) was detected after two doses of modRNA hTERT treatment (n=4). *p<0.05; One-way ANOVA or Two-way ANOVA, Dunnett's or Sidak's multiple comparisons test.

### Supplementary Figure 3| Validation of circular RNA.

**A** Validation of the size of produced linear and circular RNA products using IVT encoding for hTERT or GFP. To prove superior stability of circular RNA the linear and circular RNAs were treated with RNase R and loaded on 1.5 % denatured gel. Circular GFP is generated by substituting hTERT ORF with the GFP coding sequence. **B** Validation of circularization by performing PCR with divergent Primer of reverse transcribed circular RNA. Loaded on 1.5 % agarose gel. **C** Representative images confirming successful transfection of circular RNA GFP 2 µg/mL in HEK293 cells. **D** No significant increase observed in caspase 3/7 activity between linear and circular hTERT RNA products at 24 h. Caspase 3/7 activity returned back to baseline at 48 h indicating no adverse effect on cell viability (n = 3). Two-way ANOVA, Dunnett's multiple comparisons test.

### Supplementary Figure 4| Overexpression of hTERT mRNA after modRNA hTERT transfection in PCLS.

**A** Schematic presentation of PCLS (precision-cut lung slices) preparation. PF: pulmonary fibrosis. **B** Fold change of hTERT mRNA expression after 48 h of transfection with modRNA hTERT (1 µg/mL) compared to modRNA GFP (modRNA control) (1 µg/mL) in PCLS at 24 h (n = 2).  **C** Protein expression levels of cytokines (IFNγ, IL1β, IL2, IL4, IL6, IL8, IL10, IL12p70, IL13 and TNFα) relative to total expression after RNA hTERT treatment compared to modRNA GFP (linear RNA control) (n = 3). **D** TGFβ and pro-COL1A1 protein levels at 96 h relative to total expression after RNA hTERT treatment compared to modRNA GFP (linear RNA control) (n = 3). *p<0.05; **p<0.01; ***p<0.001; One-way ANOVA or Two-way ANOVA, Dunnett's multiple comparisons test.
